# Predictive value of adipose to muscle area ratio based on MRI at knee joint for postoperative functional outcomes in elderly osteoarthritis patients following total knee arthroplasty

**DOI:** 10.1186/s13018-020-02014-9

**Published:** 2020-10-27

**Authors:** Guanglei Zhao, Changquan Liu, Kangming Chen, Feiyan Chen, Jinyang Lyu, Jie Chen, Jingsheng Shi, Gangyong Huang, Yibing Wei, Siqun Wang, Jun Xia

**Affiliations:** Department of Orthopedics, Huashan Hospital, Fudan University, Shanghai, 200040 China

**Keywords:** Adipose to muscle area ratio (AMR), Body mass index (BMI), Osteoarthritis (OA), Predictor, Patient-reported outcome measures (PROMS), Total knee arthroplasty (TKA)

## Abstract

**Background:**

The current research used a new index—adipose to muscle area ratio (AMR)—to measure fatness compared with body mass index (BMI) in elderly osteoarthritis (OA) patients following total knee arthroplasty. Our study aimed to test the relationship between the two indexes (AMR and BMI) and to examine whether AMR was a predictive factor of patient-reported outcome measures (PROMS) for elderly OA patients following total knee arthroplasty (TKA).

**Methods:**

The retrospective data of 78 OA patients (older than 60 years) following TKA was included in our study. Clinical features of patients included age, BMI, sex, AMR, side of the implant, time of follow-up, complications, the Knee Society Score (KSS score), and the Hospital for Special Surgery knee score (HSS score). The area of adipose tissue and muscle tissue was measured on the cross section (supra-patella, midline of the patella, joint line of the knee) of the knee magnetic resonance imaging (MRI). AMR was calculated as the average of adipose to muscle area ratio at the three levels. The Pearson correlation analysis, simple linear regression, and multiple linear regression were used to study the relationship between BMI, AMR, and PROMS (KSS total-post score and HSS-post score) in the study.

**Results:**

Of all patients, the mean (± standard deviations (SD)) of age was 67.78 ± 4.91 years. For BMI and AMR, the mean (± SD) were 26.90 ± 2.11 and 2.36 ± 0.69, respectively. In Pearson correlation analysis, BMI had a good correlation with AMR (*r* = 0.56, *p* = 0.000), and AMR (*r* = − 0.37, *p* = 0.001, HSS-post score; *r* = − 0.43, *p* = 0.000, KSS total-post score) had better correlations with PROMS postoperatively compared with BMI (*r* = − 0.27, *p* = 0.019, HSS-post score; *r* = − 0.33, *p* = 0.003, KSS total-post score). In multivariate linear regression analysis, AMR was negatively correlated with KSS total-post score as well as HSS-post score, while BMI was not. As for patients with complications, AMR values were between the 3rd quartile and 4th quartile of the AMR value in the entire study cohort.

**Conclusions:**

In this study, the new obesity evaluation indicator—AMR, which was well related with BMI, was found to be a predictor of PROMS (KSS total-post score and HSS-post score) in elderly OA patients following TKA.

## Introduction

Obesity is a worldwide health problem and has almost tripled since 1975 throughout the world [1]. According to a 2016 data, there were 43.2 million men and 46.4 million women in China, and the number of obese people exceeded that of the USA, ranking first in the world [2]. As a health problem, obesity is more common in elderly people and is associated with many diseases, including osteoarthritis (OA) [3–6].

As we all know, body mass index (BMI) is currently the common indicator used to evaluate obesity and BMI ≥ 30 kg/m^2^ is defined as obesity [7]. There were many studies showing that obesity (high BMI) was associated with poor outcomes of elderly OA patients following total knee arthroplasty (TKA) [8–12]. Two meta-analyses showed that the risk of both shallow and deep infections was higher in obese patients who have underwent TKA than that of non-obese patients [8, 9]. Another meta-analysis made by Sun et al. [10] proposed that high BMI influenced postoperative functional outcomes and enhanced the risk of complications in patients following TKA. However, using BMI alone to assess the prognosis in elderly OA patients may have certain limitations. BMI is not an absolute health indicator because it does not provide a specific proportion of body composition such as the muscle, fat, and bones [13]. Sarcopenic obesity, often seen in elderly patients, in which total muscle mass, muscle strength, and physical functional decrease, could not be well represented through BMI [14, 15]. Sometimes barely relying on BMI may lead to classification bias and delay surgery for obese patients [16]. Patients with central obesity may have thin limbs and they are likely to have a good prognosis after TKA. However, such patients usually have higher BMI [17]. Meanwhile, there were studies reporting that no significant correlation was found between BMI and functional outcomes in elderly OA patients after TKA [18, 19].

To address the limitations of BMI in assessing prognosis in elderly OA patients following TKA, there were many researches using other indicators (fat mass, skeletal muscle mass, subcutaneous fat thickness, knee mass index, and so on) to better provide body composition in obese patients and study their relationship with patients’ prognosis [17, 20, 21]. It was worth mentioning that, in a recent study, Dai et al. [22] proposed a new indicator—adipose to muscle area ratio (AMR)—to assess fatness and they concluded that compared with BMI, AMR at the knee joint showed better predictive ability in predicting functional outcomes for patients following meniscectomy.

Obese patients were more susceptible to OA than those who were not, and TKA was an end-stage treatment for elderly OA patients [3, 23, 24]. AMR proposed by Dai et al. [22] was a new index to assess obesity and showed good predictive ability of postoperative outcomes in patients undergoing meniscectomy. There was no other study using AMR to assess obesity and examine its predictive ability of prognosis in elderly OA patients undergoing TKA. Our research is the first study trying to use this new index to evaluate obesity and test its predictive ability of postoperative outcomes in elderly OA patients after TKA. And we want to answer three questions through our research: (1) Is AMR at knee joint related to BMI used to evaluate obesity? (2) Can AMR at knee joint be used as a predictor of postoperative reported outcomes (the Knee Society Score (KSS score) and the Hospital for Special Surgery knee score (HSS score)) for elderly OA patients following TKA, and is it better than BMI? (3) Is AMR a predictor in predicting complications of elderly OA patients undergoing TKA?

## Methods

### Study design and study cohort

**Fig. 1:**
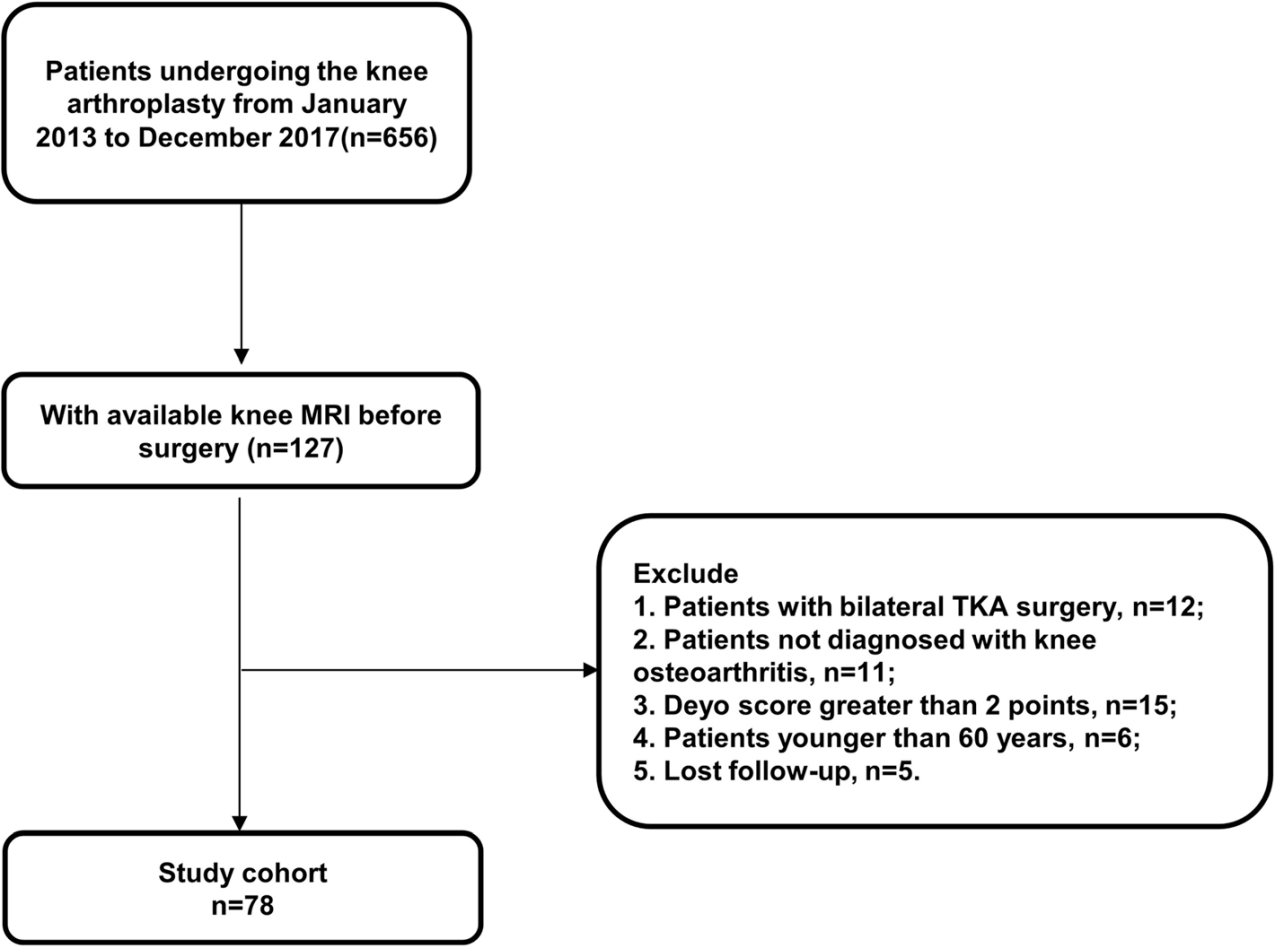
Flow diagram of the selecting process in the study

We retrospectively analyzed the total knee replacement cases (*n* = 656) in our hospital (Huashan Hospital, Fudan University) from 2013 to 2017. Among all the patients, we only retained those with knee magnetic resonance imaging (MRI) before surgery (*n* = 127). Then we excluded 49 patients according to the exclusion criteria: (1) patients with bilateral TKA surgery (*n* = 12), (2) patients not diagnosed with knee OA (*n* = 11), (3) Deyo score greater than 2 points (*n* = 15), (4) patients younger than 60 years (*n* = 6), and (5) lost to follow-up (*n* = 5). Finally, we included a total of 78 patients in our study. Figure 1 shows the screening process for the study. This study was approved by the institutional review board of Huashan Hospital, Fudan University.

The clinical features included age, BMI, sex, adipose to muscle area ratio (AMR), side of the implant, time of follow-up, and complications. The KSS score and the HSS score were used in our study to assess the functional outcome of patients. The KSS score, which is a questionnaire designed to evaluate the knee of patients, includes two parts: a knee score and a function score, both of which score from 0 to 100, with high scores representing better status [25]. The HSS score, which ranged from 0 to 100 points, contained a total of 7 parts (pain—30 points, functional—22 points, activity—18 points, muscle strength—10 points, knee flexion—10 points, stability—10 points, and point reduction) [26, 27]. Both the KSS score and HSS score were recorded before surgery (KSS-pre and HSS-pre) and at the most recent follow-up (KSS-post and HSS-post).

### Measurements of AMR

**Fig. 2:**
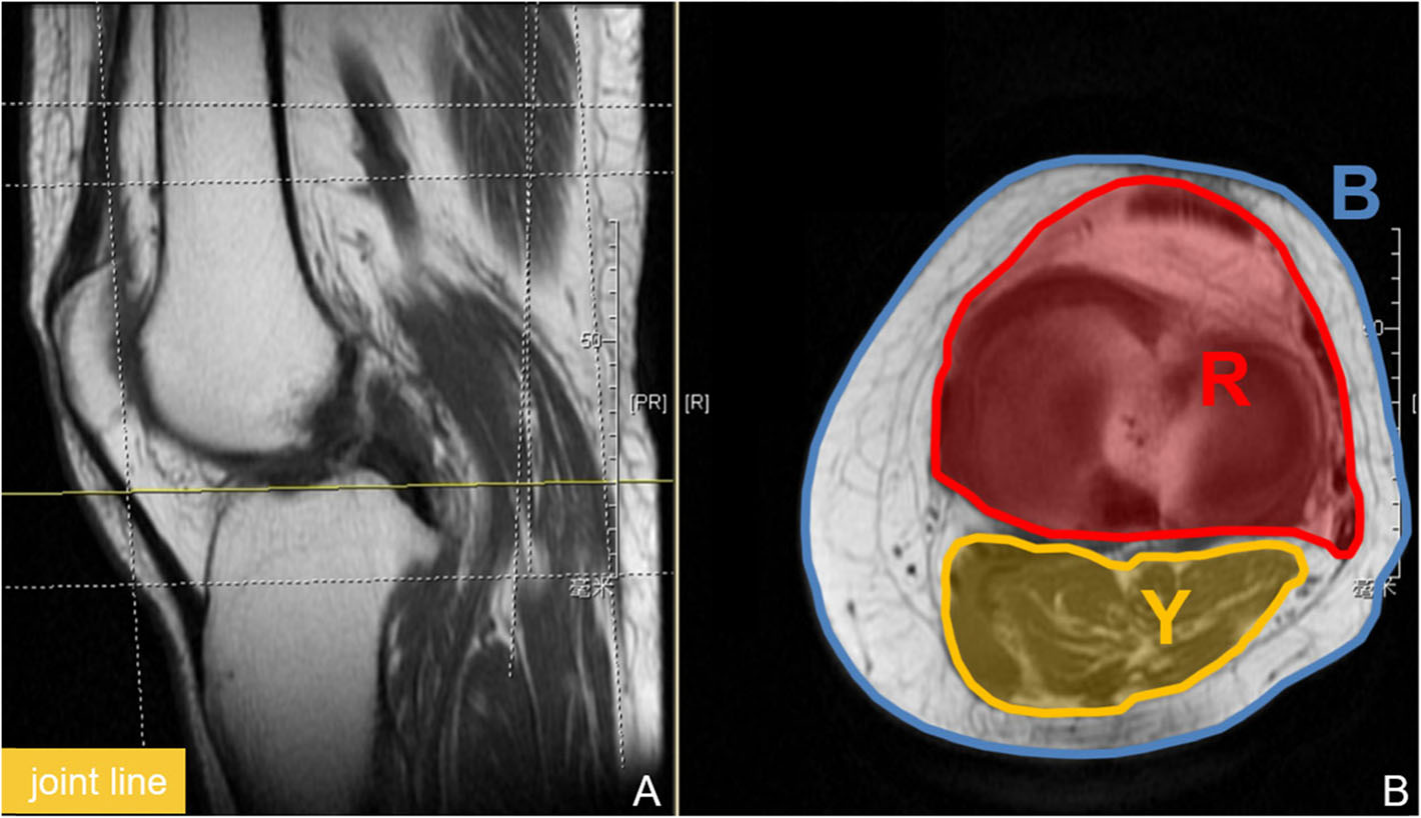
**a**, **b** Schema of AMR measurement on the cross section of the knee MRI at the level of the knee joint line. **a** Sagittal plane. **b** Cross section. The area of the joint cavity (R), the muscle tissue (Y), and the entire cross section (B)

A method used to calculate AMR at the level of the joint line was introduced in a previous study by Dai et al. [22]. In our study, the area of adipose tissue and muscle tissue was measured on the cross section of the knee MRI (supra-patella, midline of the patella, joint line of the knee). The red part was the area of the joint cavity (R), the yellow part was the area of the muscle tissue (Y), the part circled by the blue line was the area of the entire cross section (B), and the area of the adipose tissue (A) was calculated by subtracting Y and R from B. AMR = A/Y (single level), AMR at the level of the supra-patella was recorded as AMR up, AMR at the level of the midline of the patella—AMR middle, and AMR at the joint line level—AMR down. The average of the AMRs at the three levels was taken as the final AMR (recorded as AMR in our study) (Fig. 2). Area measurement was done with the software ImageJ. Good inter-observer reliability of AMR measurements was shown between two observers using the intraclass correlation coefficient (ICC) (ICC-AMR up = 0.982, ICC-AMR middle = 0.991, ICC-AMR down = 0.984).

### Complications

Composite complications after TKA included superficial incisional surgical site infection (SSI), deep incisional SSI, organ space SSI, wound disruption, pneumonia, unplanned intubation, pulmonary embolism, on ventilator > 48 h, progressive renal insufficiency, acute renal failure, urinary tract infection, stroke/cerebrovascular accident (CVA), coma > 24 h, peripheral nerve injury, dislocation, pseudoaneurysm, cardiac arrest, myocardial infarction, prosthesis failure, implant loosening, deep vein thrombosis (DVT), high metal ion levels of blood, sepsis, septic shock, and so on [28–35].

### Statistical analysis

The continuous variables (age, BMI, AMR, time of follow-up, KSS score, and HSS score) were presented as means and standard deviations (SD), while the categorical variables (sex, side of the implant) were given as frequencies and percentages (%).

Student’s *t* test was carried out to examine the discrepancy of AMR value in different genders and sides of the implant. The Pearson correlation analyses were done among those variables (age, AMR, BMI, HSS-pre, and KSS total-pre). Correlations between AMR and KSS total-post, AMR and HSS-post, BMI and KSS total-post, and BMI and HSS-post were also analyzed using the same method. The correlation coefficient (*r*) was used to measure the correlation between two variables. Positive values represented positive correlations while negative correlations were revealed by negative values. The larger the absolute value, the stronger the correlation (|*r*| = 1 represents a linear relationship). In simple linear regression analysis, variables KSS total-post and HSS-post score were defined as dependent variables, while age, BMI, sex, AMR, side of the implant, KSS total-pre score, and HSS-pre score were analyzed as independent variables. Variables (*p* < 0.1) in univariate analysis were further analyzed in multivariate linear regression analysis.

Student’s *t* test, the Pearson correlation analysis, simple linear regression analysis, and multivariate linear regression analysis were conducted in SPSS 24.0. All analyses with *p* < 0.05 (two-sided) were considered statistically significant.

## Results

In our study, a total of 78 patients were included. Of all patients, the mean (± SD) of age was 67.78 ± 4.91 years, the BMI 26.90 ± 2.11 kg/m^2^, the AMR 2.36 ± 0.69, the follow-up time 31.91 ± 6.49 months, the KSS total-pre score 105.68 ± 20.84, the KSS total-post score 181.51 ± 11.05, the HSS-pre score 47.77 ± 8.34, and the HSS-post score 91.74 ± 4.77. In this study, 43 patients were female and 35 patients were male. The surgical sites of 43 patients were the right knee and the remaining 35 patients were the left knee. A total of three patients had postoperative complications: one had a superficial incisional surgical site infection (SSI), and another two had a urine tract infection and a prosthesis failure (periprosthetic infection), respectively (Tables 1 and 5). As for AMR values, no significant difference was found between male and female, not in sides of the implant, either.

**Table 1:** Baseline characteristics

Parameter	Continuous variables: mean ± SD; categorical variables: frequency (%)
Age (years)	67.78 ± 4.91
BMI (kg/m^2^)	26.90 ± 2.11
Sex	
Male	35 (44.9%)
Female	43 (55.1%)
AMR	2.36 ± 0.69
Side	
Right	43 (55.1%)
Left	35 (44.9%)
Follow-up (months)	31.91 ± 6.49
KSS score	
KSS knee-pre	49.14 ± 9.92
KSS function-pre	56.54 ± 11.47
KSS total-pre	105.68 ± 20.84
KSS knee-post	89.91 ± 5.23
KSS function-post	91.60 ± 6.77
KSS total-post	181.51 ± 11.05
HSS score	
HSS-pre	47.77 ± 8.34
HSS-post	91.74 ± 4.77

*SD* standard deviation, *BMI* body mass index, *AMR* adipose to muscle area ratio, *KSS score* the Knee Society Score, *HSS score* the Hospital for Special Surgery knee score

In Pearson correlation analysis, significant correlations between AMR and BMI (*r* = 0.56, *p* = 0.000), age and KSS total-pre (*r* = 0.230, *p* = 0.042), and HSS-pre and KSS total-pre (*r* = 0.919, *p* = 0.000) were shown. Meanwhile, AMR had stronger correlations with PROMS (KSS total-post and HSS-post score) compared with BMI (Table 2).

**Table 2:** Correlation analysis between AMR and BMI, AMR and KSS total-post, AMR and HSS-post, BMI and KSS total-post, and BMI and HSS-post

	BMI		KSS total-post		HSS-post	
	*r*	*p*	*r*	*p*	*r*	*p*
AMR	0.56	**0.000**	− 0.43	**0.000**	− 0.37	**0.001**
BMI	–	–	− 0.33	**0.003**	− 0.27	**0.019**

*BMI* body mass index, *AMR* adipose to muscle area ratio, *KSS score* the Knee Society Score, *HSS score* the Hospital for Special Surgery knee score

In the simple linear regression analysis of PROMS (KSS total-post score and HSS-post score), AMR (*p* = 0.000) and BMI (*p* = 0.003) were negatively related with the KSS total-post score. It was the same two variables (AMR, *p* = 0.001; BMI, *p* = 0.019) showing negative correlations with the HSS-post score. In further multivariate analysis, only AMR was investigated to have a significant relationship with the KSS total-post score (*p* = 0.007), as well as with the HSS-post score (*p* = 0.014), while BMI was not (Tables 3 and 4).

**Table 3:** Simple and multiple linear regression of HSS-post score

	Simple linear regression			Multiple linear regression		
	*β*	95% CI	*p*	*β*	95% CI	*p*
Age	0.043	− 0.179 to 0.265	n.s			
BMI	− 0.599	− 1.096 to − 0.102	**0.019**	− 0.166	− 0.742 to 0.410	n.s
Sex	− 0.361	− 2.551 to 1.828	n.s			
AMR	− 2.587	− 4.054 to − 1.120	**0.001**	− 2.223	− 3.990 to − 0.455	**0.014**
Side	0.727	− 1.458 to 2.912	n.s			
KSS total-pre	0.046	− 0.005 to 0.097	**0.078**	0.018	− 0.105 to 0.141	n.s
HSS-pre	0.115	− 0.013 to 0.243	**0.078**	0.060	− 0.248 to 0.368	n.s

*CI* confidence interval, *n.s* nonsignificant, *BMI* body mass index, *AMR* adipose to muscle area ratio, *KSS score* the Knee Society Score, *HSS score* the Hospital for Special Surgery knee score

**Table 4:** Simple and multiple linear regression of KSS total-post score

	Simple linear regression			Multiple linear regression		
	*β*	95% CI	*p*	*β*	95% CI	*p*
Age	0.021	− 0.494 to 0.535	n.s			
BMI	− 1.740	− 2.867 to − 0.614	**0.003**	− 0.727	− 2.028 to 0.574	n.s
Sex	0.153	− 4.924 to 5.230	n.s			
AMR	− 6.817	− 10.134 to − 3.500	**0.000**	− 5.573	− 9.564 to − 1.582	**0.007**
Side	3.159	− 1.867 to 8.814	n.s			
KSS total-pre	0.069	− 0.052 to 0.189	n.s			
HSS-pre	0.235	− 0.063 to 0.533	n.s			

*CI* confidence interval, *n.s* nonsignificant, *BMI* body mass index, *AMR* adipose to muscle area ratio, *KSS score* the Knee Society Score, *HSS score* the Hospital for Special Surgery knee score

The interquartile range (IQR) of the AMR for the entire study cohort was 1.83–2.72. In the study, we found that the AMR values of patients with complications were between 75 and 100% of the values of the entire study cohort (3rd quartile–4th quartile). The AMR values of patients with complications were 2.80 (superficial incisional surgical site infection (SSI)), 3.15 (urinary tract infection), and 3.63 (prosthesis failure-periprosthetic infection), respectively (Table 5).

**Table 5:** Relationship between AMR and complications following TKA

	Complications^1^
AMR 1st quartile (bottom 25%) (0.85–1.83)	0
AMR 2nd quartile (1.83–2.27)	0
AMR 3rd quartile (2.27–2.72)	0
AMR 4th quartile (top 25%) (2.72–5.56)	3

^1^1 superficial incisional surgical site infection (SSI), AMR − 2.80; 1 urinary tract infection, AMR − 3.15; 1 prosthesis failure (periprosthetic infection), AMR − 3.63

## Discussion

In our research, we used a new indicator—AMR—which was well correlated with BMI to evaluate obesity in elderly OA patients following TKA. This indicator was found to be a predictor of PROMS (HSS-post score and KSS total-post score), while BMI was not. This illustrated that AMR in the knee joint was a stronger risk factor of poor functional outcomes more than BMI. Moreover, patients with complications were investigated with high AMR values (range: 3rd quartile to 4th quartile) in our study, which suggested that AMR might have a correlation with postoperative complications in elderly OA patients after TKA.

BMI had many advantages in measuring obesity of elderly OA patients, such as ease of use and accurately defining obesity in most cases [13]. Many studies had showed there was a correlation between BMI and outcomes for elderly OA patients following TKA [8, 9, 11, 36]. Xu et al. [36] found that patients with higher BMI were more likely to have a smaller enhancement in functional outcome scores (Oxford Knee Score (OKS)). Giesinger et al. [11] found that BMI had a negative impact on functional outcome scores and satisfaction scores for patients following TKA. Although a lot of articles investigated that BMI negatively affected postoperative outcomes in elderly OA patients after TKA, there was no agreement on this issue [18, 19]. Overgaard et al. [18] reported that the 1-year functional outcomes of patients following TKA were not influenced by BMI in a cohort of 3327 patients. Sveikata et al. [19] found no significant influence of BMI on postoperative functional outcome (OKS) for patients undergoing TKA. In a multivariate linear analysis of our study, no significant relationships between BMI and PROMS (KSS total-post score and HSS-post score) were investigated (Tables 3 and 4). These results showed the limitations of BMI to assess adiposity and evaluate the prognosis in elderly OA patients following TKA. The limitations of BMI were due to its own characteristics [13, 16]. People with the same BMI may have different organizational ratios. Using BMI alone to assess the postoperative outcomes in elderly OA patients following TKA ignored the effects of different body components on patients’ prognosis, especially the muscle mass component. In contrast to BMI, AMR could better show the local distribution of muscle mass and adipose components in the knee joint. Meanwhile, the strength of quadriceps was related to the prognosis of patients after TKA. These might explain that AMR had stronger correlations with PROMS compared with BMI (Table 2) and could be used as a predictor of functional outcomes in elderly patients following TKA (Tables 3 and 4) in our research. To note, there were studies proposing that extremities of BMI might have better predictive ability of functional outcomes after surgery [22, 37]. Therefore, we speculated that AMR might be a supplement to BMI in body composition evaluation, especially in the assessment of local extremities.

Sarcopenic obesity was defined as a state in which obesity and sarcopenia co-occur. This term was described as total muscle mass, muscle strength, and physical functional decline often in the elderly [14, 15, 38, 39]. EWGSOP recommends using computed tomography (CT), magnetic resonance imaging (MRI), dual-energy X-ray absorptiometry (DXA), and bioimpedance analysis (BIA) to measure muscle mass [14]. Therefore, AMR measured through MRI can be regarded as one of sacropenia’s evaluation methods. In recent studies, Babu et al. [15] found that the psoas-lumbar vertebral index (measured by CT images), an indicator of central sacropenia, had good predictive value for prosthetic joint infections (PJIs). Chang et al. [40] investigated that paraspinal muscle density (PSD) and skeletal muscle index (SMI) at L4 (an indicator of sacropenia) had negative effects on perioperative outcome for patients who had proximal femur fractures. Wagner et al. [41] discovered that larger psoas cross-sectional area (CSA) was a protective factor of degenerative spondylolisthesis. In line with them, we found AMR was an independent predictor of PROMS (KSS total-post score and HSS-post score) in elderly OA patients following TKA in our study (Tables 3 and 4). Meanwhile, the current use of MRI was becoming more and more widespread in process of diagnosis and treatment [42–45]. These results to some extent supported the feasibility of AMR in the knee joint to be a predictor of functional outcomes in elderly OA patients after TKA.

For a long time, obesity has often been found to be associated with complications (shallow infection, deep infection, pulmonary embolism, etc.) in elderly OA patients following TKA [8, 9, 46, 47]. Using meta-analysis, Kerkhoffs et al. [8] and Si et al. [9] found that the risk of both shallow and deep infections was higher in obese patients following TKA. DeMik et al. [46] and Sloan et al. [47] reported that the chance of postoperative complications would increase in obese patients following TKA. In our research, we used the new indicator—AMR—to evaluate obesity and found that patients with complications had higher AMR value of the entire study cohort (3rd quartile–4th quartile) (Table 5). Such an observation prompted that patients with high AMR might be more prone to postoperative complications.

### Limitations

Our research has its limitations. First, the patients in our study had only functional outcome measurements. There was no physical and mental health assessment for patients following TKA due to the shortage of our database. However, KSS and HSS scores used in our research were representative in assessing the postoperative condition of TKA patients and could reflect the patients’ clinical results well. Second, AMR in our study was a local parameter (surgical site). The muscle area in the measurement site only included part of the quadriceps (closely related to the knee joint). We failed to select the middle femur (the quadriceps were more developed) for research due to the limitations of knee MRI. Nevertheless, our research improved the measurement method of AMR by Dai et al. [22]. In our study, we measured AMR value in three levels of the knee (supra-patella, midline of the patella, joint line of the knee) and used their average as final AMR, with the hope to objectively reflect the local status of muscle and fatness of the knee. Third, the measurement and calculation of AMR were based on MRI, while MRI was not a routine examination before TKA in all patients. Nevertheless, as MRI had become a common examination in the diagnosis and treatment of knee joint diseases, this new indicator—AMR—could, to some extent, help doctors to predict the patient’s prognosis and to make more precise treatment and rehabilitation plans. Finally, the number of samples was too small and the conclusions that originated from our research should be used with caution. It was important to point out that there were only 3 patients with postoperative complications, so that we could not use statistical methods to test the relationship between the AMR value and postoperative complications. However, based on the limited patients, we found AMR was a predictor of PROMS and might have a relationship with postoperative complications for the patients in our study. If there were more large-scale studies in the future, the effectiveness of AMR could be better verified.

## Conclusions

In this study, the new obesity evaluation indicator—AMR, which was well related with BMI, was found to be a predictor of PROMS (KSS total-post score and HSS-post score) in elderly OA patients following TKA, while BMI was not. This suggested that AMR in the knee joint might be used as a stronger risk factor of functional outcomes in elderly OA patients after TKA more than BMI.

## Data Availability

All the data are available in contact with the correspondence author.

## Abbreviations

AMR: Adipose to muscle area ratio
BMI: Body mass index
OA: Osteoarthritis
PROMS: Patient-reported outcome measures
TKA: Total knee arthroplasty
KSS score: The Knee Society Score
HSS score: The Hospital for Special Surgery knee score
MRI: Magnetic resonance imaging
SD: Standard deviations
SSI: Surgical site infection
CVA: Cerebrovascular accident
DVT: Deep vein thrombosis
IQR: Interquartile range
CT: Computed tomography
DXA: Dual-energy X-ray absorptiometry
BIA: Bioimpedance analysis
PJIs: Prosthetic joint infections
PSD: Paraspinal muscle density
SMI: Skeletal muscle index
CSA: Cross-sectional area
